# Study on the standardization method of radiotelephony communication in low-altitude airspace based on BART

**DOI:** 10.3389/fnbot.2025.1482327

**Published:** 2025-04-02

**Authors:** Weijun Pan, Boyuan Han, Peiyuan Jiang

**Affiliations:** ^1^Civil Aviation Flight University of China, Chengdu, China; ^2^University of Electronic Science and Technology of China, Chengdu, China

**Keywords:** radiotelephony communication, air traffic control, BART model, low-altitude airspace, deep reinforcement learning

## Abstract

The development of air traffic control (ATC) automation has been constrained by the scarcity and low quality of communication data, particularly in low-altitude complex airspace, where non-standardized instructions frequently hinder training efficiency and operational safety. This paper proposes the BART-Reinforcement Learning (BRL) model, a deep reinforcement learning model based on the BART pre-trained language model, optimized through transfer learning and reinforcement learning techniques. The model was evaluated on multiple ATC datasets, including training flight data, civil aviation operational data, and standardized datasets generated from Radiotelephony Communications for Air Traffic Services. Evaluation metrics included ROUGE and semantic intent-based indicators, with comparative analysis against several baseline models. Experimental results demonstrate that BRL achieves a 10.5% improvement in overall accuracy on the training dataset with the highest degree of non-standardization, significantly outperforming the baseline models. Furthermore, comprehensive evaluations validate the model’s effectiveness in standardizing various types of instructions. The findings suggest that reinforcement learning-based approaches have the potential to significantly enhance ATC automation, reducing communication inconsistencies, and improving training efficiency and operational safety. Future research may further optimize standardization by incorporating additional contextual factors into the model.

## 1 Introduction

### 1.1 Introduction

As of the end of 2023, there were 259 transportation airports within mainland China (excluding Hong Kong, Macau, and Taiwan), 690 traditional general aviation enterprises with general aviation operating licenses, and a total of 3,303 registered general aviation aircraft, including 1,398 aircraft used for flight training. The number of registered and managed general airports nationwide reached 449, of which 163 were classified as Class A general airports. From these data, it can be seen that China’s civil aviation transportation has developed very rapidly, especially the development of general aviation, which has led to more complex operations in low-altitude airspace (Civil Aviation Administration of China, 2024).

Air traffic control plays a crucial role in ensuring aviation safety and enhancing flight efficiency. During the control process, controllers send instructions to pilots via very high frequency (VHF) radio, and pilots must accurately repeat these instructions to ensure correct understanding (Lin et al., 2020). Although radiotelephony communications for air traffic control have unique and stringent regulations (Lin et al., 2019a; Geacăr, 2010), in practice, the dialogues between controllers and pilots often loosely follow standardized phrases. This is because they frequently add colloquial terms and make simple errors, leading to a high degree of arbitrariness and non-standardization in control instructions. This not only increases the complexity of communication but also elevates the risk of misunderstandings and errors. This phenomenon is especially prevalent in the control of low-altitude airspace, typically managed by approach and tower controllers. Furthermore, the rise of the “low-altitude economy” has prompted diverse stakeholders—from local governments to private enterprises—to explore the use of low-altitude airspace for cargo transportation, tourism, emergency response, and other commercial activities. Because these activities frequently rely on smaller airports or temporary landing areas near urban or suburban zones, the command structures involved become more decentralized, and the personnel managing low-altitude traffic can come from varied backgrounds, not always under the direct supervision of civil aviation authorities. These factors introduce additional variability and risk into radiotelephony communications. According to regulations by the International Civil Aviation Organization (2020) and Civil Aviation Administration of China (2015), controllers at high-traffic airports are required to undergo additional training to adapt to busy and complex airspace environments. Consequently, a standardized communication model capable of adapting to such settings is crucial for ensuring safety and efficiency in future low-altitude economic activities.

In recent years, significant progress has been made in the application of artificial intelligence (AI) technology in the aviation field, especially in air traffic control, flight plan optimization, and aviation safety monitoring. However, widespread use in frontline operations is challenging, with most implementations being small-scale experimental setups (Glaser-Opitz and Glaser-Opitz, 2015; Lin et al., 2019b; Serdyuk et al., 2018). AI technology, through methods such as big data analysis, machine learning, and deep learning, can enhance the automation of aviation systems, reduce human errors, and improve overall operational efficiency and safety. Nevertheless, the effectiveness of AI systems relies on a large amount of high-quality data. In the field of air traffic control, the scarcity and confidentiality of data pose substantial challenges to the promotion and application of AI technology.

For small and medium-sized airports, the issue of data scarcity is particularly prominent. Due to resource and scale limitations, these airports find it difficult to obtain a large amount of standardized training data. Additionally, individual communication habits vary, and in radiotelephony communications training, the apprenticeship model is used, resulting in differences in communication habits even among different shifts at the same airport. This further increases the variance in the data. The lack of standardized data not only affects the training effectiveness of AI models but also increases errors in actual applications due to instruction discrepancies.

In summary, it is crucial to research how to improve the model’s ability to handle non-standard texts and its robustness. The BRL model proposed in this paper converts non-standard control texts into standardized texts. This approach can help trainees familiarize themselves with and master standardized phrases in control simulators, improving training effectiveness. It can also expand the standardized data sets of small and medium-sized airports, enhancing the generalization and adaptability of AI models. Additionally, real-time text standardization technology can assist controllers and pilots in understanding and executing instructions more clearly, reducing communication misunderstandings and errors. Overall, this research provides an effective solution to the highly diverse data distribution encountered in low-altitude airspace due to varying communication habits and regional differences. By bolstering the model’s generalizability across diverse operational scenarios, it further facilitates the broader adoption of AI in the field of air traffic control, thereby enhancing operational safety and training efficiency. Building upon this foundation, the key contributions of this paper are as follows:

1. Model design and proposal: a standardization model based on the BART framework combined with deep reinforcement learning (BART-Reinforcement Learning, BRL) was proposed. This approach enhances the robustness of BART’s text generation against interference and noise while improving the model’s generalization capability.
2. Innovative intent-based evaluation method: to better assess the standardization of air-ground communication instructions, dedicated evaluation metrics were designed for different intent categories, including Command and Readback Accuracy (CRA), Fixed Coordination Reporting Accuracy (FCRA), Non-fixed Coordination Reporting Accuracy (NCRA), Other Text Accuracy (OA), and Total Accuracy (TA). Compared to traditional ROUGE metrics, this approach provides a more comprehensive evaluation of the model’s standardization effectiveness.

### 1.2 Related work

Non-standard radiotelephony communications text normalization refers to the process, in radiotelephony communications scenarios, of refining redundant, non-standard, or colloquial utterances and converting them into accurate and standardized phraseology to ensure efficient and safe information transmission. In essence, it belongs to one of the text generation tasks. Text generation tasks typically include machine translation, text summarization, and dialogue generation: machine translation focuses on equivalence across different languages, text summarization aims to extract or generate the core information of the source text in a concise manner, while dialogue generation centers on producing coherent responses in an interactive context. By comparison, non-standard radiotelephony communications text normalization most closely resembles text summarization in its effort to handle redundant and colloquial content, extracting key directive elements. However, it simultaneously emphasizes strict adherence to industry norms and precision in expression. Therefore, from a task-oriented perspective, it can be categorized under the text summarization branch of text generation.

In recent years, researchers have proposed a variety of models for text generation and normalization tasks, primarily including the sequence-to-sequence (Seq2Seq) model, the Transformer model, and generation architectures based on pre-trained language models. The Seq2Seq model, introduced by Sutskever et al. (2014), was initially applied to machine translation. This model employs an encoder-decoder architecture that encodes the input sequence into a fixed-length context vector, from which the decoder generates the target sequence. Bahdanau et al. (2014) further introduced the attention mechanism, enabling the model to dynamically attend to different parts of the input sequence during decoding, thereby enhancing its capability for modeling long sequences. Luong et al. (2015) investigated various types of attention mechanisms and achieved favorable results in machine translation tasks. The Seq2Seq model has the advantages of simplicity in structure, ease of implementation, and suitability for short sequence generation tasks. Moreover, it allows end-to-end training, reducing the need for extensive feature engineering. Nonetheless, its primary drawback lies in its limited ability to model long sequences: a fixed-length context vector can lead to information loss, especially when handling inputs containing multiple intentions or complex semantics, resulting in lower-quality generated text. Wiseman and Rush (2016) and Cho et al. (2014) also underscored this issue, noting that despite improvements brought by attention mechanisms, the model remains constrained in processing lengthy sentences and comprehending multiple intentions.

To overcome the limitations of Seq2Seq, Vaswani et al. (2017) proposed the Transformer model, which relies solely on self-attention mechanisms and discards recurrent neural network structures. The Transformer can capture global dependencies in the input sequence and supports parallel computation, substantially enhancing training efficiency and generation quality. Tian et al. (2020) demonstrated that the Transformer outperforms traditional Seq2Seq models in tasks such as sequence transformation and machine translation, showing significant advantages particularly in dealing with long sequences and complex sentence structures (Bapna et al., 2018). The main advantages of the Transformer model include its simple architecture, training efficiency, capacity for modeling long-range dependencies, and strong scalability. However, its drawbacks are also evident: it requires substantial computational resources, a large amount of training data, and is prone to overfitting when data are limited. These disadvantages are especially pronounced in the radiotelephony communications domain, where data are scarce due to confidentiality and where annotations must be performed by professionals. Consequently, applying the Transformer model in real-world operations becomes challenging.

In recent years, generation architectures based on Pre-trained Language Models (PLMs) have achieved remarkable progress in text generation tasks. Raffel et al. (2020) proposed the T5 model, which unifies all text-processing tasks into a text-to-text paradigm, broadening the scope of Transformer applications in text generation. BERT (Bidirectional Encoder Representations from Transformers) (Devlin et al., 2019) employs a bidirectional encoder for deep semantic understanding; although it excels in natural language understanding tasks, its lack of an autoregressive generation component limits its effectiveness in direct text generation. As an improved version of BERT, RoBERTa leverages larger-scale data and optimized training strategies to enhance understanding performance (Liu et al., 2019), yet it remains confined to an encoder architecture that is ill-suited to complex generation tasks. In contrast, the GPT (Generative Pre-trained Transformer) model (Radford et al., 2019) adopts a decoder structure and uses an autoregressive approach, demonstrating robust text generation capabilities in language modeling and dialogue generation. However, its unidirectional property renders it less effective in scenarios requiring bidirectional context comprehension. BART (Bidirectional and Auto-Regressive Transformers) (Lewis et al., 2019) combines BERT’s bidirectional encoding with GPT’s autoregressive decoding, employing a denoising auto encoder pre-training objective. This enables strong semantic understanding while generating high-quality text, achieving state-of-the-art performance across various text generation tasks. Due to the unique characteristics of radiotelephony communications—such as succinct sentence structures, diverse expressions, and the need for precise, standardized instructions—BART’s integration of bidirectional understanding and generative capabilities is particularly effective in handling non-standard inputs and producing standardized phraseology, making it the most suitable choice for this task. Despite the excellent performance of PLM-based methods in text generation, they share a common drawback: they rely on cross-entropy as the loss function, causing the generation process to hinge predominantly on Maximum Likelihood Estimation (MLE), which can lead to exposure bias and inconsistencies with evaluation metrics (Ranzato et al., 2015). Moreover, the optimization objectives in pre-trained models are typically not directly aligned with real-world evaluation metrics (e.g., BLEU, ROUGE, or semantic matching), making it difficult to guarantee the final generated text aligns perfectly with task-specific goals.

To address these challenges, we incorporate reinforcement learning (reinforcement learning, RL) (Paulus et al., 2017). RL is a machine learning framework in which an agent interacts with an environment and adjusts its policies based on feedback (rewards or penalties) to maximize cumulative reward. In text generation tasks, the model receives a reward based on semantic consistency between the generated text and the target text for each token it generates, thereby learning to produce standardized outputs. The reward mechanism inherent in RL aligns naturally with the requirement of generating text that closely matches the semantics of standardized instructions, allowing direct optimization of final generation quality rather than mere surface-level similarity. By incorporating task-specific rewards, RL can enhance the consistency of generated text and improve model stability. Paulus et al. (2017) showed that RL effectively improves alignment between generated texts and evaluation metrics in text summarization tasks, while Wu et al. (2018) demonstrated that it significantly reduces exposure bias and improves model robustness (Wang and Sennrich, 2020).

Ultimately, we combine BART with reinforcement learning to introduce task-related reward signals during generation, enabling end-to-end optimization that notably mitigates exposure bias and enhances consistency between the generated text and the target standards. This approach ensures both semantic accuracy and adherence to standard protocols in the generated output, delivering more stable and reliable performance in radiotelephony communications normalization tasks.

## 2 Methods and data

### 2.1 Methodology

Firstly, the model is pre-trained on data from relevant domains to grasp general language features and patterns. Subsequently, we apply this pre-trained model to the task of standardizing radiotelephony communications texts for air traffic control and perform fine-tuning. This method better adapts the model to the characteristics and norms of control communication texts, thereby enhancing its performance in this task; secondly, to address the issue of distribution differences in radiotelephony communications texts, the policy gradient algorithm directly optimizes the generation policy, handles complex sequence generation, adapts to diverse inputs, utilizes rich feedback signals, achieves stable policy updates, and incorporates historical information, thereby improving the quality, coherence, and robustness of the generated texts; finally, due to the specificity and professionalism of radiotelephony communications, we introduce a new evaluation criterion based on control intent to assess the quality of the generated radiotelephony communications texts. This evaluation criterion more accurately reflects the model’s performance in the task of standardizing control communication texts and provides valuable guidance for model improvement.

### 2.2 Deep reinforcement learning architecture

Deep reinforcement learning, as a method that combines deep learning and reinforcement learning, is widely applied to solve decision-making problems with high-dimensional state and action spaces. It uses deep neural networks as function approximators, playing a crucial role in the end-to-end learning process from raw input to action selection. In the task of text summarization, the application of deep reinforcement learning provides a new approach to model training, enabling the generation of higher quality summaries. Its core framework involves an agent learning the optimal policy through interaction with the environment. The agent observes the current text state and selects the action of generating the next word based on a reward function. By continually optimizing the policy network parameters to maximize cumulative rewards, the summarization process is optimized. The key lies in designing appropriate state representations, action spaces, reward functions, and policy networks to ensure that the model effectively learns text semantics and contextual information, thereby generating higher quality summaries.

**Figure 1:**
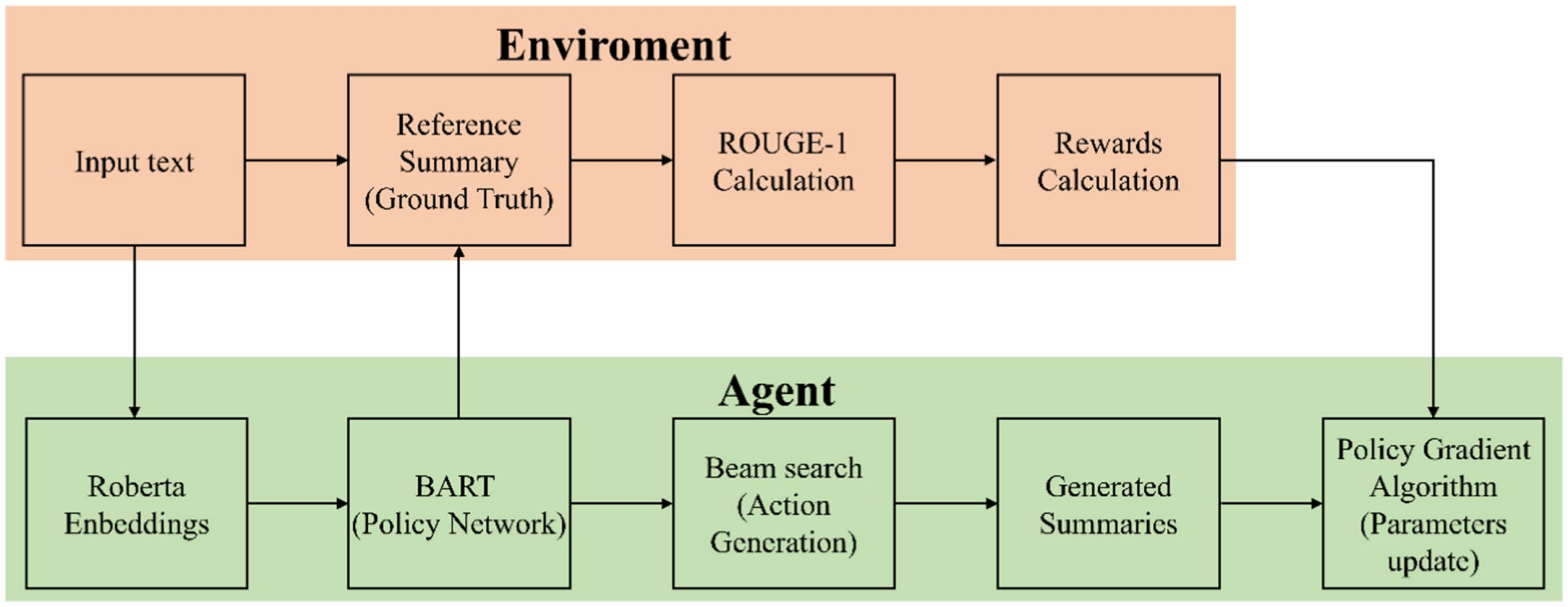
Deep reinforcement learning model architecture.

The model uses the BART model to encode the raw text and employs the BART model as the policy network. Through Beam Search, it generates candidate texts and uses the ROUGE-1 score as a reward function to evaluate the quality of the generated texts. The policy gradient algorithm is used to optimize the parameters of the BART model, gradually improving the standardization of the generated texts. Through these steps, the model can efficiently process and standardize radiotelephony communications texts, enhancing the model’s generalization capability. The deep reinforcement learning model architecture we propose is shown in Figure 1.

Figure 1 details the training process of the proposed deep reinforcement learning model architecture. First, the input raw X is encoded using the BART model, converting it into the state representation S , as described by the Equation 1:


(1) $$S=\mathrm{BARTEmbedding}\left(X\right)$$

Using the BART model, candidate target texts Y are generated from $\pi_{\theta }$ , as described by the Equation 2:


(2) $$Y=\pi_{\theta }\left(S\right)$$

Using the Beam Search algorithm, multiple candidate texts are generated from the BART model, and the highest-scoring $Y^{*}$ is selected, as described by the Equation 3:


(3) $$Y^{*}=\mathrm{Beamsearch}\left(\pi_{\theta }\left(S\right)\right)$$

The ROUGE-1 score between the generated text $Y^{*}$ and the reference standard text $Y_{ref}$ is calculated as the reward r , as described by the Equation 4:


(4) $$r=\mathrm{ROUGE}-1\left(Y^{*},Y_{ref}\right)$$

The loss function $L\left(\theta \right)$ approximates the expected value by averaging over the training samples, where |D| represents the number of samples in the experience replay buffer, $r_{i}$ represents the reward for the i -th sample, $Y_{i}^{*}$ represents the best target text for the i -th sample, and $S_{i}$ represents the embedding of the non-standard text for the i -th sample, as described by the Equation 5:


(5) $$L\left(\theta \right)=-\frac{1}{\left|D\right|}\overset{\left|D\right|}{\underset{i=1}{\sum }}r_{i}\cdot \log\pi_{\theta }\left(Y_{i}^{*}|S_{i}\right)$$

The BART model parameters θ are updated using the gradient descent method, where α represents the learning rate, as described by the Equation 6:


(6) $$\theta =\theta -\alpha \nabla_{\theta }L\left(\theta \right)$$

### 2.3 Experimental data

The experiment comprised three datasets: a training flight radiotelephony communication recording text dataset consisting of 4,910 entries from fourteen hours of flight, a civil aviation frontline air traffic control command recording text dataset containing 9,414 entries, and a dataset of 1,000 generated standardized control texts. The generated standardized control text dataset was edited based on the industry standard “Radiotelephony communications for air traffic services” (MH/T 4014-2003) issued by the Civil Aviation Administration of China. In the following text, the civil aviation frontline air traffic control command recording text dataset is referred to as the operational text, the generated standardized control text dataset is referred to as the generated text, and the training flight radiotelephony communication text dataset is referred to as the training text. The pre-trained model has been trained on a large-scale corpus comprising both Chinese and English texts, endowing it with robust bilingual language understanding capabilities. Building upon this foundation, we have employed reinforcement learning techniques, specifically Reinforcement Learning from Human Feedback (RLHF), to fine-tune the model’s outputs, ensuring they align closely with human expectations. This process enables the model to adeptly generate English words and sentences that meet specified requirements. Consequently, the model demonstrates exceptional performance in handling both Chinese and English content, alleviating any concerns regarding its bilingual proficiency.

After carefully studying the industry standards issued by the Civil Aviation Administration of China and the radiotelephony communication scenarios in actual operations, it was found that there are some humanistic nuances included when implementing these standards. For example, certain units may specify not abbreviating “ILS approach” as “ILS” while neighboring units may not have such a regulation. Therefore, the definition of standardization for the same text may vary across different units. To ensure that the standardization in this experiment better reflects the actual situation, three experienced frontline controllers were invited to annotate the standardized texts. One of them is a supervisor with twelve years of experience in training flight command at Suining Nanba Airport, and he has also participated in civil aviation air traffic control command at Nanchong Gaoping Airport. The second one is a controller with four years of experience at Lhasa International Airport, responsible for tower and approach control at Lhasa Airport. The third one is a controller with four years of experience in approach control at Nanchang. Based on the practical work experience of these controllers and the industry standard “Radiotelephony communications for air traffic services” we annotated the standardized texts for both the training and operational texts.

During the experiment, since the generated control texts were entirely standardized, we expanded these texts by randomly selecting some and introducing common errors made by controllers during actual command processes. Our augmentation involved three types of errors, with each selected text incorporating 1 to 2 of the following:

1. Random Insertion of Colloquial Words: Inserting colloquial expressions such as “Ah” and “Um” to simulate natural speech patterns. For example “Em Air China four four two one climb to Um six hunderds meters.”
2. Correction Command Errors: Simulating scenarios where controllers repeat or correct instructions without using the term “correction,” which can lead to misunderstandings. For example “Air China four four two one descend to six hundred meters.”

In the end, the training data consisted of 4,710 entries of training text, 9,214 entries of operational text, 9,214 entries of augmented operational text, and 900 entries of generated text. The testing data included 200 entries of training text, 200 entries of operational text, 200 entries of augmented recording text, and 100 entries of generated text Table 1 presents some examples from the datasets.

**Table 1:** Examples of air traffic control instruction text datasets.

Dataset instance	Non-standard text	Standard text
Training text	One Zero Whiskey Uniform after baoshengzhen, direct to penglai, climb to one thousand eight hundred meters, One Zero Whiskey Uniform	One Zero Whiskey Uniform after baoshengzhen, direct to penglai, climb to one thousand eight hundred meters
Operational text	China Eastern offset change to five miles right of track	China Eastern offset five miles right of track
Augmented operational text	China Eastern uh offset change to uh five miles right of track	China Eastern offset five miles right of track
Generated text	Air China eight six seven four contact PinLiang one one one um decimal one eight	Air China eight six seven four contact PinLiang one one one decimal one eight

**Figure 2:**
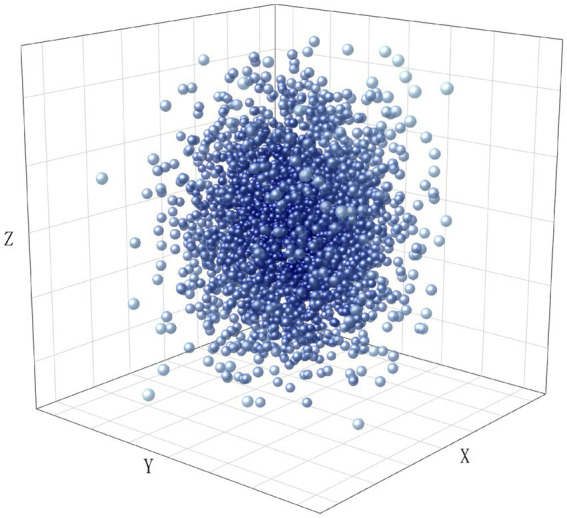
The distribution of word vectors for generated text.

**Figure 3:**
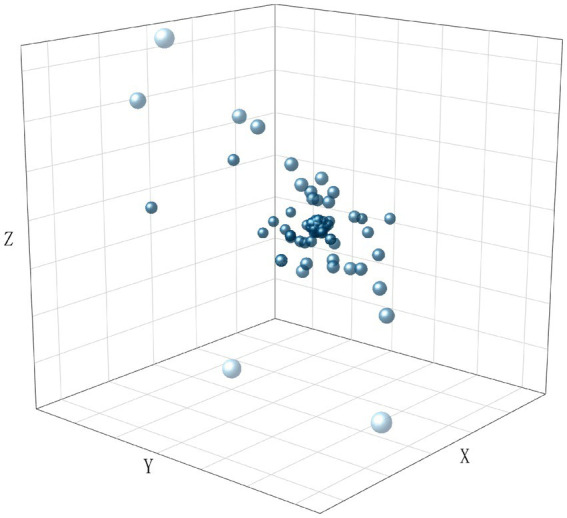
The distribution of word vectors for training text.

**Figure 4:**
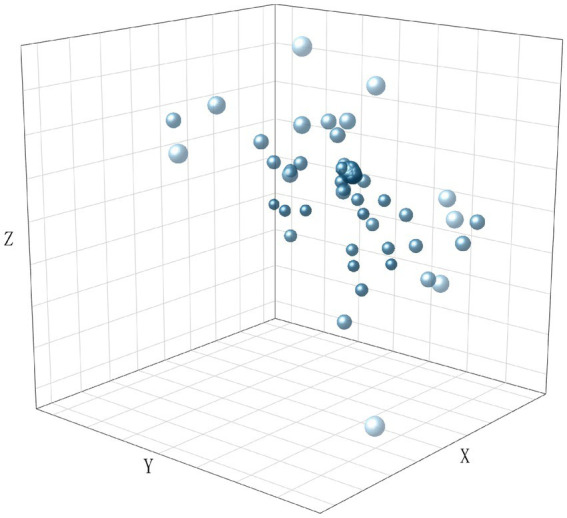
The distribution of word vectors for augmented operational text.

**Figure 5:**
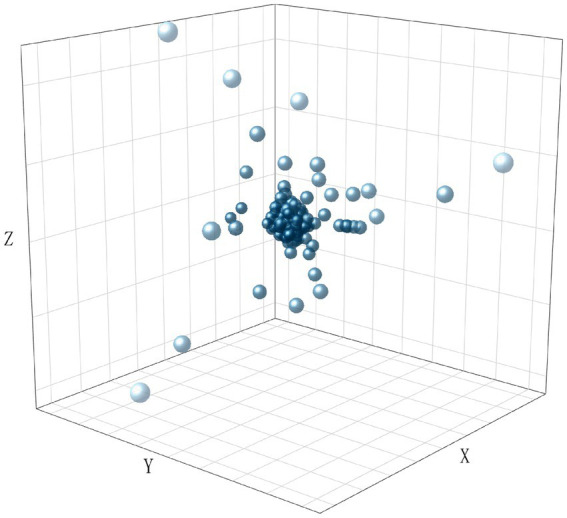
The distribution of word vectors for operational text.

To visualize the distribution of the dataset, we encoded all texts using TF-IDF and performed dimensionality reduction using LSA. The results are shown in Figures 2–5, in the reduced-dimensional data, the X, Y, and Z axes represent the directions with the greatest variance in the data. From the figures, it can be observed that the distribution of generated text is the most uniform, as it follows a fixed format. Augmented operational text exhibits a more dispersed distribution compared to the operational text, while operational text and training text show similar levels of dispersion. This is because operational text consists of independent single command instructions, whereas training text contains complete training conversation texts with context, encompassing a variety of coordination and command content, leading to more complex and diverse text content with uneven word vector distributions.

### 2.4 Evaluation methodology

ROUGE (Recall-Oriented Understudy for Gisting Evaluation) is a set of metrics used to evaluate automatically generated summaries or machine translation results (Thomas et al., 2022). The advantage of the ROUGE evaluation method lies in its simplicity and effectiveness in measuring the similarity between generated texts and reference texts. It is widely applicable to tasks such as automatic summarization, machine translation, and text generation. The specific ROUGE metrics include: ROUGE-N: Calculates overlaps based on n-grams (e.g., ROUGE-1, ROUGE-2). ROUGE-L: Considers sequence and global matching information based on the Longest Common Subsequence (LCS). ROUGE-W: Uses a weighted longest common subsequence, emphasizing continuous matches. ROUGE-S: Based on skip-bigrams, allowing for non-continuous matches (Lin, 2004).

For evaluating the effectiveness of standardizing radiotelephony communications texts, the advantages of ROUGE-N and ROUGE-L lie in their ability to meticulously assess word and phrase matches while considering order and global structure. This makes them well-suited for evaluating the results of standardizing radiotelephony communications. The drawbacks of ROUGE-W and ROUGE-S are that while they provide more flexibility and matching possibilities, their complexity and potentially overly lenient matching standards may reduce their effectiveness in tasks requiring strict semantic and structural matches (Lin and Och, 2004).

The calculation method for ROUGE-N is Equation 7:


(7) $$\mathrm{ROUGE}-N=\frac{\underset{S\in \mathrm{referencesummaries}}{\sum }\underset{gram_{n}\in S}{\sum }\mathrm{Coun}t_{\mathrm{match}}\left(gram_{n}\right)}{\underset{S\in \mathrm{referencesummaries}}{\sum }\underset{gram_{n}\in S}{\sum }\mathrm{Count}\left(gram_{n}\right)}$$

In the translation you provided, n represents the length of n-grams, $\mathrm{Coun}t_{\mathrm{match}}\left(gram_{n}\right)$ denotes the maximum count of n-grams that appear simultaneously in the generated summary and the corresponding reference summary, while $\mathrm{Count}\left(gram_{n}\right)$ indicates the count of n-grams in the reference summary.

The calculation method for ROUGE-L is Equations 8–10:


(8) $$R_{LCS}=\frac{LCS\left(X,Y\right)}{len\left(Y\right)}$$

(9) $$P_{LCS}=\frac{LCS\left(X,Y\right)}{len\left(X\right)}$$

(10) $$F_{LCS}=\frac{\left(1+\beta^{2}\right)R_{LCS}P_{LCS}}{R_{LCS}+\beta^{2}P_{LCS}}$$

Among these, $R_{LCS}$ represents the recall rate, $P_{LCS}$ represents the precision rate, and $F_{LCS}$ is the ROUGE-L value. The parameter β is adjustable and used to balance the focus between recall and precision. If the focus is more on recall, it implies that we want the generated text to include as much information from the reference text as possible. A high recall rate indicates that the important information in the reference text is well reflected in the generated text. Conversely, if the focus is more on precision, it implies that we want the generated text to be more accurate, reducing the introduction of irrelevant information. A high precision rate indicates that most of the information in the generated text is important information from the reference text.

In contrast to everyday conversations, air traffic control (ATC) communications exhibit stronger norms, although this does not imply that there’s only one correct way to phrase each instruction. ATC communications require language to be concise and precise, avoiding hesitant words like “um” or “uh.” In actual operational scenarios, some content can be omitted without affecting the completeness of instructions. Therefore, using only the ROUGE evaluation method may not comprehensively assess the model’s performance in generating standardized texts. For example, in the training flight dataset, the original recording states “contact Wuhan one one two DAY-SEE-MAL two thank you for your guidance one two niner Delta,” while the standardized form is “contact Wuhan one one two DAY-SEE-MAL two goodday one two niner Delta”, THE model’s output is “contact Wuhan one one two DAY-SEE-MAL two goodday thank you for your guidance one two niner Delta”, which, if evaluated using the ROUGE series, would yield the evaluation results as shown in the table. However, according to ATC regulations, the model’s output instructions are also correct and standardized, even more so than the original instructions.

From the ROUGE series evaluation results in Table 2, it is evident that while the ROUGE evaluation method can generally reflect the model’s ability to generate text, it cannot comprehensively assess the model’s capability to generate standardized air traffic control (ATC) communications. Therefore, considering the characteristics of ATC communications and the varied normative requirements across different units, in addition to manually evaluating the quality of text generation, a new evaluation method has been established based on the intent of ATC instructions.

**Table 2:** ROUGE series evaluation results of examples.

Evaluation method	Number of n-grams in standard instructions	Number of overlapping n-grams between model output and standard	Results
ROUGE-1	12	10	0.833
ROUGE-2	11	9	0.818
ROUGE-L	12	10	0.833

First, this study categorize all air traffic control (ATC) communications into four types, including: command and readback texts, fixed coordination report texts, non-fixed coordination report texts, and other texts. Specifically, command and readback texts refer to instructions issued by controllers that require pilots to execute tasks, as well as the texts where pilots read back these instructions. Fixed coordination report texts refer to coordinated instructions with fixed formats, such as controllers reporting flight dynamics. Non-fixed coordination report texts refer to unstructured coordination and reporting, such as pilots reporting weather conditions encountered, or controllers reporting sudden situations, as well as altitude coordination between controllers and pilots. Other texts refer to texts other than the above categories, which generally do not affect ATC communications, such as “hello” and “thank you.” We have annotated each communication text with all intent categories it contains.

During radio communication, the most common types of texts are command and readback texts, followed by fixed coordination report texts. These two types of texts are required to strictly adhere to communication rules. If the standardized text does not strictly follow these rules or contains typographical errors or omissions, it will be marked as incorrect. In contrast, non-fixed coordination report texts and other texts only need to maintain semantic consistency to be considered correct. Therefore, during the standardization process, these two types of texts are generally not altered. When conducting statistics, correctness is marked based on semantic accuracy. In the statistics, if a text contains an error in any part, it is counted. For multiple errors of the same type within a single text, we count only once. Finally, we calculate the proportion of correct texts in each category.

Based on the above, the evaluation indicators based on the intent of the instruction include the following four types: CRA (Command and Readback Text Accuracy), FCRA (Fixed Coordination Reporting Text Accuracy), NCRA (Non-fixed Coordination Reporting Text Accuracy), OA (Other Text Accuracy). The specific calculation formula as Equation 11:


(11) $$A{\mathrm{ccurate}}_{\mathrm{type}}=\frac{\sum_{t\in T_{\mathrm{type}}}C\left(t\right)}{\left|T_{\mathrm{type}}\right|}$$

### 2.5 Experimental setup and results

The experiments were carried out on a Windows operating system with the following computer configuration: an Intel Core i5-8400 processor, 56GB of RAM, NVIDIA RTX 4090 24GB graphics card, 250GB SSD, and a 3.6 TB HDD. The deep learning framework utilized was PyTorch. Table 3 presents the hyperparameter settings for the BRL model.

**Table 3:** Model architecture hyperparameters.

Hyperparameters	Values
Dropout	0.1
Max sequence length	128
Learning rate	0.0001
Batch size	16
Number of epoch	20
Optimizer	Adam
Beamsearch	3
Weight_decay	0.001

## 3 Results

To assess the performance of our constructed model, we conducted comparative studies with current mainstream pre-trained models, including GPT-2, BERT, Roberta and Bart.

**Figure 6:**
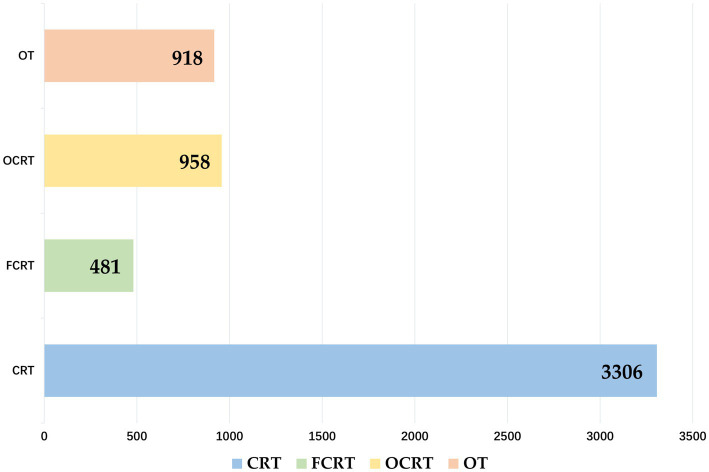
Distribution of intent in training texts.

To better explore the effectiveness of the models across various distributions, we annotated the intent types of all training texts, as shown in Figure 6. Thus, when testing the models on the three datasets, both the ROUGE series evaluation methods and human evaluation employed for assessment. Additionally, for the training text, we conducted evaluation using an intent-based evaluation method for air-ground communication commands. Experimental results are presented in Tables 4–9.

**Table 4:** The results of GPT2 based on the ROUGE and MER evaluation standard.

GPT2				
Evaluation methodology	Training text	Operational text	Augmented operational text	Generated text
ROUGE-1	0.933	0.977	0.979	0.929
ROUGE-2	0.864	0.970	0.971	0.828
ROUGE-L	0.932	0.977	0.979	0.929
MER	0.790	0.960	0.965	0.050

**Table 5:** The results of BERT based on the ROUGE and MER evaluation standard.

BERT				
Evaluation methodology	Training text	Operational text	Augmented operational text	Generated text
ROUGE-1	0.832	0.970	0.959	0.974
ROUGE-2	0.687	0.955	0.923	0.952
ROUGE-L	0.823	0.970	0.959	0.974
MER	0.285	0.890	0.630	0.730

**Table 6:** The results of BART based on the ROUGE and MER evaluation standard.

BART				
Evaluation methodology	Training text	Operational text	Augmented operational text	Generated text
ROUGE-1	0.934	0.980	0.980	0.988
ROUGE-2	0.872	0.976	0.974	0.983
ROUGE-L	0.933	0.980	0.980	0.988
MER	0.800	0.980	0.970	1

**Table 7:** The results of Roberta based on the ROUGE and MER evaluation standard.

Roberta				
Evaluation methodology	Training text	Operational text	Augmented operational text	Generated text
ROUGE-1	0.929	0.977	0.978	0.987
ROUGE-2	0.859	0.977	0.972	0.983
ROUGE-L	0.928	0.977	0.978	0.987
MER	0.745	0.980	0.980	1

**Table 8:** The results of BRL based on the ROUGE and MER evaluation standard.

BRL				
Evaluation methodology	Training text	Operational text	Augmented operational text	Generated text
ROUGE-1	0.941	0.979	0.981	0.988
ROUGE-2	0.873	0.973	0.974	0.983
ROUGE-L	0.940	0.979	0.981	0.988
MER	0.865	0.980	0.960	1

**Table 9:** Results comparing based on instruction intent evaluation criteria for training texts.

Model	CRA	FCRA	NCRA	OA	TA
GPT2	0.805	0.800	0.555	0.941	0.790
BERT	0.309	0.150	0.148	0.411	0.285
BART	0.834	0.800	0.555	0.882	0.800
RoBERTa	0.762	0.800	0.518	0.882	0.745
BRL	0.907	0.900	0.667	0.941	0.905

To evaluate the performance of our constructed model, we conducted a comparative study with the current mainstream pretrained models, including GPT-2, BERT, Roberta and Bart. As the generated text and operational text are independent single-line command texts, while the training text consists of complete training communication texts with context, the training text is comparatively less standardized and contains various types of coordination and command content, we have annotated the intent types for all training texts, and the results are shown in the Figure 6. Therefore, when testing each model on the three datasets, we employed both the ROUGE series evaluation method and manual evaluation, for the training text, we further utilized an evaluation method based on the intent of air traffic control (ATC) instructions. The experimental results are shown in Tables 4–9.

According to the results in Tables 4–8, from the perspective of the Rouge evaluation method, all the control models perform well on the operation text and generated text tests, with our proposed BRL model slightly outperforming the control models. However, it is worth noting that on the training text dataset, the generalization performance of all control models is not ideal, exhibiting significant generalization problems, while the performance of our proposed model only slightly decreases. Table 9 provides a detailed display of the performance of each model under the evaluation criteria based on instruction intent. It can be observed that our constructed BRL model demonstrates the best performance on all datasets, indicating that our proposed improvement strategy greatly alleviates the problem of poor generalization in transfer learning. These performance disparities primarily stem from each model’s underlying architecture. GPT-2, leveraging autoregressive mechanisms with unidirectional attention, excels in generating coherent text but may introduce redundancies or omissions in scenarios that demand thorough context comprehension and adherence to specialized ATC conventions. BERT and RoBERTa, although adept at semantic encoding via bidirectional context modeling, have relatively limited sequence-decoding capabilities; thus, when strict formatting is required, they can produce errors in key fields or overall consistency. Moreover, ATC instruction texts are characterized by a “highly standardized” core format while still allowing for a certain degree of colloquial or variant expressions. Such conditions require models not only to preserve precise structure but also to handle and correct potential deviations. BRL addresses these needs by incorporating reinforcement learning into BART’s encoder–decoder framework, where metrics such as ROUGE serve as reward functions to iteratively refine alignment with standardized references. Through repeated optimization, BRL more effectively resolves potential errors and maintains semantic consistency, allowing it to balance the rigorous requirements of ATC instructions with the flexibility to accommodate minor textual variations. Consequently, BRL demonstrates superior stability and generalization across diverse ATC datasets, further validating its effectiveness in this task.

## 4 Conclusion

This study developed a standardized instruction generation method for real-time radiotelephony communications using a deep reinforcement learning model, BRL, based on the BART pre-trained language model. The BRL model demonstrated substantial improvements in standardizing control instructions across multiple datasets, including training flight data, civil aviation control operation data, and generated control instructions derived from the “Radiotelephony communications for air traffic services” standard.

Our method effectively addresses the challenges of non-standard communication practices prevalent in air traffic control, particularly in low-altitude control scenarios managed by small and medium-sized airports. By leveraging transfer learning and reinforcement learning techniques, the BRL model achieved certain improvements under the ROUGE evaluation criteria and demonstrated significant performance enhancements under the evaluation criteria based on the intent of control instructions, with an overall accuracy increase of 12% compared to the baseline model.

The comprehensive evaluation, including a novel assessment based on the intent of land-air communication instructions, highlighted the model’s capability to enhance the clarity and correctness of instructions, thereby reducing communication misunderstandings and errors. This advancement not only improves training effectiveness for air traffic controllers but also enhances the operational efficiency and safety of real-time air traffic control communications.

The findings suggest that the implementation of such AI-driven models can significantly mitigate the generalization challenges of transfer learning models across disparate datasets, promoting broader adoption and application of AI technology in air traffic control. Due to the aviation industry’s stringent requirements for accuracy and speed, large-scale models do not meet practical needs; therefore, we have not considered such approaches. Future work will focus on further refining the model and exploring its application in other domains requiring standardized communication protocols.

## Data Availability

The datasets presented in this study can be found in online repositories. The names of the repository/repositories and accession number(s) can be found at: https://drive.google.com/drive/folders/1lqVwCDVZInXsz-BqnVxcsAFL87lz8hTL?usp=drive_link.
